# Anaerobic HgII reduction is driven by cellular HgII-thiol interactions

**DOI:** 10.1099/acmi.0.000932.v3

**Published:** 2025-01-28

**Authors:** N.C. Lavoie, A.J. Poulain

**Affiliations:** 1 Department of Biology, Faculty of Sciences, University of Ottawa, Ottawa, Canada

**Keywords:** bacteria, fermentation, mercury, phototrophy, redox, thiol

## Abstract

Redox reactions play a critical role in determining the availability of mercury species, Hg^II^ and Hg^0^, to anaerobic microbes responsible for methylating inorganic mercury into toxic monomethylmercury. Some anaerobes also contribute to Hg cycling in methylation hotspots by reducing Hg^II^ to its gaseous elemental form, Hg^0^. However, their contributions remain poorly quantified due to limited mechanistic insights and the absence of genetic targets. In this study, we investigated the mechanisms of anaerobic Hg^II^ reduction in the versatile anoxygenic photoheterotroph and fermenter *Heliomicrobium modesticaldum* Ice1. Given Hg^II^ strong electrophilic affinity for thiol groups, we hypothesized that cellular thiols are key interaction sites mediating Hg^II^ reduction. Exposure of *H. modesticaldum* to the thiol-alkylating agent *N*-ethylmaleimide (NEM), which irreversibly binds thiols, resulted in a concentration-dependent inhibition of Hg^0^ production during both photoheterotrophy and fermentation. Hg partitioning assays with *Escherichia coli* cells revealed no significant differences in Hg-cell partitioning in the presence or absence of NEM, suggesting that Hg^II^ reduction is dependent on intracellular thiol interactions. These findings highlight the importance of thiol-mediated pathways in Heliobacterial Hg^II^ reduction. Although the exact cellular components remain unidentified, we discuss potential thiol-containing coupling sites that warrant further investigation.

## Data Summary

The authors confirm that all supporting data are provided in the article and in the supplementary data files.

## Introduction

Mercury, a toxic heavy metal, is globally distributed via atmospheric transport and can bioaccumulate and biomagnify in food webs, particularly in its methylated form, monomethylmercury (MMHg) [1]. Microbial processes heavily regulate much of Hg’s fate [2]. Its most neurotoxic form, MMHg, is produced from inorganic Hg by anaerobic micro-organisms in terrestrial and aquatic environments [3,7]. Within these anoxic environments, microbially mediated redox cycling can affect the speciation and availability of Hg substrate to methylators [8]. Its role is twofold: Hg^0^ oxidation supplies dissolved Hg^II^ required for methylation, and Hg^II^ reduction can lead to remobilization of gaseous Hg^0^. Anoxygenic phototrophs and anaerobes thrive in low light anoxic conditions also inhabited by Hg methylators. In these environments, abiotic Hg^II^ photoreduction is nearly absent; thus, anoxygenic phototrophs adapted to low light intensity and chemotrophic anaerobes stand to be critical drivers of Hg^II^ reduction [9,11] and influence MMHg formation.

Unlike many aerobes that reduce Hg via dedicated enzymatic machinery as part of a detoxification strategy encoded by the *mer* operon [12,13], genetic determinants have yet to be found for anaerobic Hg^II^ reducers [9,11,14]. The information currently available suggests that core components of anaerobic respiration [15], fermentation [9] or anoxygenic phototrophy [10] are driving Hg^II^ reduction. However, without detailed mechanisms or genetic targets, it remains challenging to assess the contribution of anaerobic Hg^II^ reduction to global Hg cycling. The purpose of our study was to gain insights into the mechanisms allowing *Heliobacteria*, a metabolically versatile anaerobe, to reduce Hg^II^ to its gaseous elemental form, Hg^0^.

*Heliobacteria* are terrestrial, spore-forming, strictly anaerobic bacteria capable of photoheterotrophy in the presence of light and fermentation in the dark [20]. They are the only known family of phototrophs from the *Bacillota* phylum, formerly *Firmicutes*. They conduct a primitive form of photosynthesis characterized by a unique photosynthetic pigment, bacteriochlorophyll g [21,22]. Its most well-studied representative, the type strain *Heliomicrobium modesticaldum* Ice1, originates from hot spring soils in Iceland and has also been found in hot spring microbial mats in Yellowstone [23].

In an early effort to investigate the diversity of phototrophs capable of anaerobic Hg^II^ reduction, we tested *H. modesticaldum* Ice1 and found that it could reduce over 80% of Hg^II^ to Hg^0^ [9]. We then discovered that another member of this family, *Heliobacterium mobile*, shared this ability [11] and that the magnitude of Hg^II^ reduced by *H. mobile* could be increased by growing it on more reduced sulphur sources such as thiosulphate. No apparent dedicated Hg^II^ reduction machinery (i.e. *mer* operon genes) was found in either of these strains. We pursued experiments with *H. modesticaldum* to further investigate the mechanism at play. In the absence of any oxidizable carbon source in the growth media, phototrophic cells could reduce Hg^II^, but fermentative cells could not. This indicates a connection between intracellular availability of reduced redox cofactors and Hg^II^ reduction and led us to investigate the role of reducing power-generating enzymes on Hg^II^ reduction. Pyruvate:ferredoxin oxidoreductase (PFOR) is one of the key carbon metabolism enzymes in *Heliobacteria*, active during both fermentative and photoheterotrophic growth [24]. It couples the oxidation of pyruvate (the carbon source) into acetyl-CoA to the reduction of ferredoxins, small one-electron-carrying redox cofactors. PFOR is thus one of the primary generators of reducing power during Heliobacterial fermentation. During phototrophy, cells can generate reducing power via PFOR activity but also via the photosynthetic electron transport chain (pETC). Both PFOR and the photosynthetic reaction centre transfer electrons to ferredoxins, which then shuttle electrons to other enzymes [24,28]. We inhibited PFOR activity in fermentative cells using the chemical nitazoxanide, which eliminated fermentative Hg^II^ reduction completely [9]. We made the same observation in another fermentative but non-phototrophic *Bacillota*, *Clostridium acetobutylicum*, as well as in *Geobacter sulfurreducens* PCA [9]. Importantly, PFOR inhibition did not affect Hg^II^ reduction under phototrophic conditions. These findings led us to propose that Hg^II^ reduction is dependent on the cells’ ability to generate reducing power, i.e. to reduce redox cofactors through the pETC or organic carbon oxidation via PFOR. Although these findings suggest a source of electrons fuelling Hg^II^ reduction, we did not identify specific enzymatic coupling points. We suspect thiol-bearing intracellular proteins or low molecular mass molecules as probable sites involved in Hg^II^ reduction on the basis of the following: (1) the reduction of Hg^II^ by *Heliobacteria* appears to be cytosolic [9,11, 29], (2) Hg^II^ is highly electrophilic and binds preferentially to SH- cysteine residues on protein surfaces [30] and (3) as reducing power availability appears to dictate the magnitude of Hg^II^ reduced [9,10], enzymes in the oxidoreductase class and their electron carriers may be involved in this metal reduction reaction.

We hypothesize that SH- are likely binding sites involved in Hg^II^ reduction. We predict that blocking Hg^II^ access to SH- groups will decrease Hg^II^ reduction. We thus searched for potential chemical inhibitors with specificity to SH- groups. *N*-Ethylmaleimide (NEM) is an organic compound that is reactive towards thiols and is commonly used to modify cysteine residues in proteins. The thiolate anion (RS^−^) of cysteine attacks the electrophilic centre of the C=C bond of the maleimide group to form a thioether bond (R-S-R) between the thiol and the maleimide [31,32]. This reaction with thiols occurs near pH 6.5–7.5, and the resulting thioether bond is virtually irreversible [31]. In the early days of discovering the mercuric reductase enzyme (MerA), researchers used NEM on an Hg^II^-reducing *Escherichia coli* strain to determine if the reduction mechanism required interaction with thiol groups for the activity to occur [33]. In their work, NEM bound to cell thiols and resulted in the inhibition of the Hg^II^ reduction phenotype in a concentration-dependent manner. It was later confirmed that the reduction activity was attributable to MerA, a flavin disulphide oxidoreductase [33,35]. NEM has also been used in studies investigating the protein complexes that make up the pETC in plant chloroplasts. *In vitro* assays with purified pETC proteins demonstrated that NEM reacts with thiol ligands on ferredoxin:NADP+ oxidoreductase (FNR), causing it to lose its catalytic activity by preventing electron transfer from ferredoxins to FNR [36].

In the present work, we show that exposing *H. modesticaldum* Ice1 to sub-lethal concentrations of NEM inhibits anaerobic Hg^II^ reduction in a concentration-dependent manner during both photoheterotrophic and fermentative metabolisms. Hg mass balance assays further suggest that the inhibition of Hg^II^ reduction by NEM is not caused by differences in Hg^II^-cell partitioning. Our findings implicate interactions between Hg^II^ and cell thiols as an important part of anaerobic Hg^II^ reduction. We further discuss mechanistic insights into possible enzymatic partners and propose avenues of future investigation for this cryptic microbial Hg transformation.

## Methods

### Microbial culturing

#### *H. modesticaldum* Ice1

MIC assays with NEM and bioreactor experiments were conducted using the strain *H. modesticaldum* Ice1 from the DSMZ culture collection (DSM-9504). Cell cultures were handled and cultured under anaerobic conditions as described in our prior work [9].

The medium composition of PYE (pyruvaye-yeast extract) is Na-pyruvate 2.2 g l^−1^, yeast extract 0.2 g l^−1^, K_2_HPO_4_ 1 g l^−1^, MgSO_4_•7H_2_O 0.2 g l^−1^, Na_2_S_2_O_3_ 1 mM, CaCl_2_•2H_2_O 0.02 g l^−1^, (NH_4_)_2_SO_4_ 1 g l^−1^ (15.1 mM of NH_4_^+^), anoxic FeSO_4_ 20 µM, d-biotin 0.015 µg ml^−1^, vitamin B12 as cyanocobalamin 0.02 µg ml^−1^ and 1 ml of trace element solution SL6. Trace element SL6 contained ZnSO_4_•7 H_2_O 0.1 g l^−1^, MnCl_2_•4 H_2_O 0.3 g l^−1^, H_3_BO_3_ 0.3 g l^−1^, CoCl_2_•6H_2_O 0.2 g l^−1^, CuCl_2_•2H_2_O 0.01 g l^−1^, NiCl_2_•6H_2_O 0.02 g l^−1^ and Na_2_MoO_4_•2H_2_O 0.03 g l^−1^.

*H. modesticaldum* cultures were incubated at 50 °C with an incandescent light at an intensity of 80 µmol photon m^−2^ s^−1^. Depending on the metabolic conditions desired, these re-inoculations and all subsequent ones were either kept in front of the light for photoheterotrophic growth or wrapped in foil and kept in the dark for fermentative growth. For MIC experiments, cells were transferred a second time after reaching mid-exponential to start the MIC assay in 10-ml Balch tubes. For bioreactor experiments, re-inoculations were into serum bottles containing 75 ml of the same growth medium. Upon reaching mid-exponential, these 75 ml cultures were then used as a 10% (v/v) inoculum into bioreactors containing the same growth medium.

#### *E. coli* NEB5α

NEM-Hg partitioning assays were conducted with *E. coli* NEB5α (DH5α derivative of the K12 strain). Cultures were prepared under anaerobic conditions in a Coy anaerobic glovebox. Cultures were revived from −80 °C cryostocks onto fumarate-Glucose Minimal Medium (GMM) plates [37] and incubated at 37 °C in anaerobic jars. A single colony was then picked and added to liquid GMM medium [37] in an anaerobic Balch tube and grown overnight. The following day, cultures were used for NEM-Hg partitioning assays.

#### NEM MIC assays

We conducted MIC assays with *H. modesticaldum* Ice1 to identify NEM concentrations to work at for later Hg partitioning assays and bioreactor experiments. Effectively, we monitored growth curves with NEM added at different concentrations. NEM powdered reagent (99+%, Thermo Scientific Chemicals) was used to prepare working solutions and was kept away from bright light sources to prevent photodegradation per the manufacturer’s material safety data sheet. *H. modesticaldum* Ice1 was cultured as previously described, and mid-exponential photoheterotrophic and fermentative cultures were added to separate Balch tubes containing PYE growth media to a final OD 600 nm of ~0.2, which is the same cell density used for bioreactor experiments. To these Balch tubes, NEM solution was added to a final concentration of 0, 100 and 200 µM. Each condition was prepared in biological triplicates. Balch tubes were then incubated at 50 °C in the light or dark, and cell growth was measured as the OD at 600 nm (OD 600 nm) in individual Balch tubes using a spectrophotometer over the course of 48 h. Additional experiments were also later included for photoheterotrophic cultures at an NEM concentration of 1000 µM after the initial assays showed cultures tolerated lower concentrations quite well.

#### NEM-Hg partitioning assays with *E. coli*, a non-reducing strain

We developed an Hg partitioning assay to quantify THg associated with different fractions (cell, media and glass associated) under NEM exposure. *E. coli* NEB5α, rather than *H. modesticaldum*, was used to test the effect of NEM on Hg partitioning because it does not reduce Hg^II^ to Hg^0^ [9]. As the *E. coli* OD 600 nm required to retrieve a sizable pellet was ~0.4, yet the bioreactor assays are conducted at an OD 600 nm of ~0.2, we doubled the concentration of Hg and NEM that *E. coli* was exposed to compared to the concentrations used for bioreactors. Thus, we maintained equal ratios of cell OD 600 nm: Hg: NEM between the partitioning assays and bioreactor experiments, i.e. 0.400 OD: 500 pM Hg: 400 µM NEM for *E. coli* assays and 0.200 OD: 250 pM: 200 µM for *H. modesticaldum* bioreactors.

In preparation for these assays, all material was acid washed for 24 h in 10% HCl to remove traces of Hg bound to glass Balch tubes, 5 ml Teflon vials or plastic 2 ml microcentrifuge tubes. All material was brought into a Coy anaerobic glovebox to deoxygenate for 48 h prior to the start of assays. Additionally, all assay steps were performed inside the anaerobic chamber.

*E. coli* was cultured as previously described. Three Balch tube cultures of similar OD 600 nm were chosen for the assay. Cultures were gently centrifuged to form pellets and resuspended twice in medium PYE to remove traces of GMM medium. In six Balch tubes with PYE medium, sufficient resuspended *E*. coli was added to reach an OD 600 nm of ~0.4 in a final volume of 10 ml. Six sterile controls containing only medium PYE were also prepared. To half of all assay Balch tubes, 400 µM of NEM solution was added; to the no NEM controls, the same volume of anoxic water was added. Tubes were wrapped in foil and then placed to incubate in a shaking incubator at 37 °C for 1 h to allow NEM to saturate cells. After 1 h, 500 pM of HgCl_2_ was added to every tube. One millilitre was subsampled from each Balch tube into microcentrifuge tubes to quantify the total Hg added at the start of the experiment. Balch tubes were then incubated for 3 h at 37 °C. We chose an assay duration of 3 h because of two lines of reasoning: (1) *E. coli-*based Hg biosensors reveal that within the first 3 h of exposure, the vast majority of Hg^II^ added can be detected intracellularly [38,39], and (2) in our anaerobic Hg^II^ reduction bioreactor assays, the first 0 to 12 h have consistently high rates of Hg^0^ production [9].

At the end of the 3-h exposure time, samples were then prepared to extract and quantify Hg associated with different fractions. To begin, we subsampled 2 ml of each live culture Balch tube into centrifuge tubes and gently spun down at 3000 r.p.m. for 10 min. After spinning down the samples, the supernatant was removed and filter sterilized through a 0.2 µM syringe filter to remove residual cells. The filtrated liquid was kept as it represents the media-associated Hg fraction. Additionally, we prepared the following blanks in Teflon tubes to quantify any background Hg from reagents: PBS buffer blank, PYE medium blank and Milli-Q blank. Next, we washed the remaining cell pellets once with 1 ml PBS buffer to remove any residual media-associated Hg, gently spun at 3000 r.p.m. for 10 min and put the discarded PBS supernatant into a microcentrifuge tube labelled ‘PBS wash’.

All microcentrifuge tubes and Balch tubes were brought to a trace metal clean lab for the next steps of the extraction. We resuspended the cell pellet with 1 ml of 70% HNO_3_, transferred the liquid to a Teflon vial and rinsed the Eppendorf tube with another 1 ml HNO_3_ to retrieve any leftover Hg. We also added the samples taken at the start of the experiment into Teflon tubes and rinsed the microcentrifuge tubes with 1 ml of 70% HNO_3_. We then put these Teflon vials in an incubator set to 55 °C to digest for 1 h. The purpose of this digestion is to break down cells and free any intracellular Hg.

Balch tubes were thoroughly emptied, and their insides were soaked for 15 min three times with 10 ml of 10% HCl to remove any Hg bound to glass, keeping 1 ml of each wash in clean Teflon tubes. Lastly, we added 1% (v/v) of 70% concentrated HNO_3_ to all Teflon vial samples not previously prepared with HNO_3_ to preserve them prior to analysis. We then prepared samples for CVAFS according to the EPA Method 1631 and quantified total Hg in samples on a Tekran® 2600 Automated Sample Analysis System.

Though we tried, we could not successfully obtain consistent intracellular versus cell-surface THg results with the *E. coli* Hg partitioning assay (data not included). The original chemical agent we used to remove cell-surface Hg, dimercaptosuccinic acid (DMSA), caused the cell pellet to lose integrity. We suspect that DMSA was destabilizing the outer membrane of *E. coli,* as reported by others [40].

#### Bioreactor experiments with *H. modesticaldum* Ice1

The bioreactor setup and subsampling methods used in this study were identical to those validated and used in prior research [9,10]. All bioreactor experiments were conducted at 50 °C in growth medium PYE. Fermentative bioreactor experiments were conducted in complete darkness, and phototrophic bioreactors were placed under a light intensity of 20 µmol photon m^−2^ s^−1^ to limit abiotic photoreduction of Hg^II^ [10]. Live cell bioreactors were performed in triplicate for each condition tested and with initial *H. modesticaldum* Ice1 cell inocula of 10% (v/v) from cultures grown until mid‐ to late‐exponential phase. An abiotic bioreactor experiment exposed to light and containing sterile PYE growth media with 100 µM NEM was performed once to confirm the absence of any NEM-mediated photoreduction of Hg^II^. For every bioreactor experiment, a fresh NEM solution was prepared by dissolving its powdered form in sterile anoxic water to a thousandfold higher concentration than the desired final concentration of NEM in the bioreactor. For example, a 200 mM stock was prepared to spike into a bioreactor for a final concentration of 200 µM. Every bioreactor experiment was spiked with 250 pM of HgCl_2_ and ran for 48 h to measure cumulative hourly Hg^0^ production using a Tekran 2537B CVAFS Analyzer. NEM solutions were always added to the bioreactor prior to spiking with Hg^II^ to allow NEM to saturate cells. Briefly, the overall order of operation for bioreactors is as follows: (1) set up the bioreactor with anoxic growth media, (2) add cell culture, (3) spike NEM stock solution into the bioreactor to reach the desired final concentration and (4) after 1 h, spike HgCl_2_ to a final concentration of 250 pM.

### Statistical analyses

#### ANOVAs

Two-way parametric ANOVAs were used to test for the effect of NEM addition on total Hg partitioning to different fractions and on cumulative Hg^0^ production during phototrophic and fermentative bioreactors. Tests for assumptions of normal distribution of residuals and constant variance were passed. For both analyses, treatments where significant differences were observed were identified using a Tukey Honest Significant Difference (HSD) post hoc test with the significance threshold set to *P*<0.05. All statistical analyses were performed in R version 4.2.2 (R Core Team) with ‘multcomp’ [41] and ‘car” [42] packages. Detailed statistical results are provided in the figure captions.

## Results

### MIC identifies a tolerable range of NEM concentration

Due to NEM’s strong cellular inhibitory potential, we first conducted MIC assays on phototrophic and fermentative cultures of *H. modesticaldum* Ice1 to identify a range of NEM concentrations to work with for subsequent experiments. In these assays, we used the same starting cell OD 600 nm as used in Hg^II^ bioreactor experiments to accurately identify a cell-to-NEM ratio to use in later bioreactor experiments. We determined that concentrations from 0 to 1000 µM of NEM had none to severe effects on growth rate and cellular yield for photoheterotrophic and fermentative cultures (Fig. S1, available in the online version of this article). Having identified a range of NEM concentrations at the cusp of causing adverse effects, we performed Hg partitioning assays at an equivalent of 200 µM and bioreactor assays at 0, 100 and 200 µM.

**Fig. 1.**
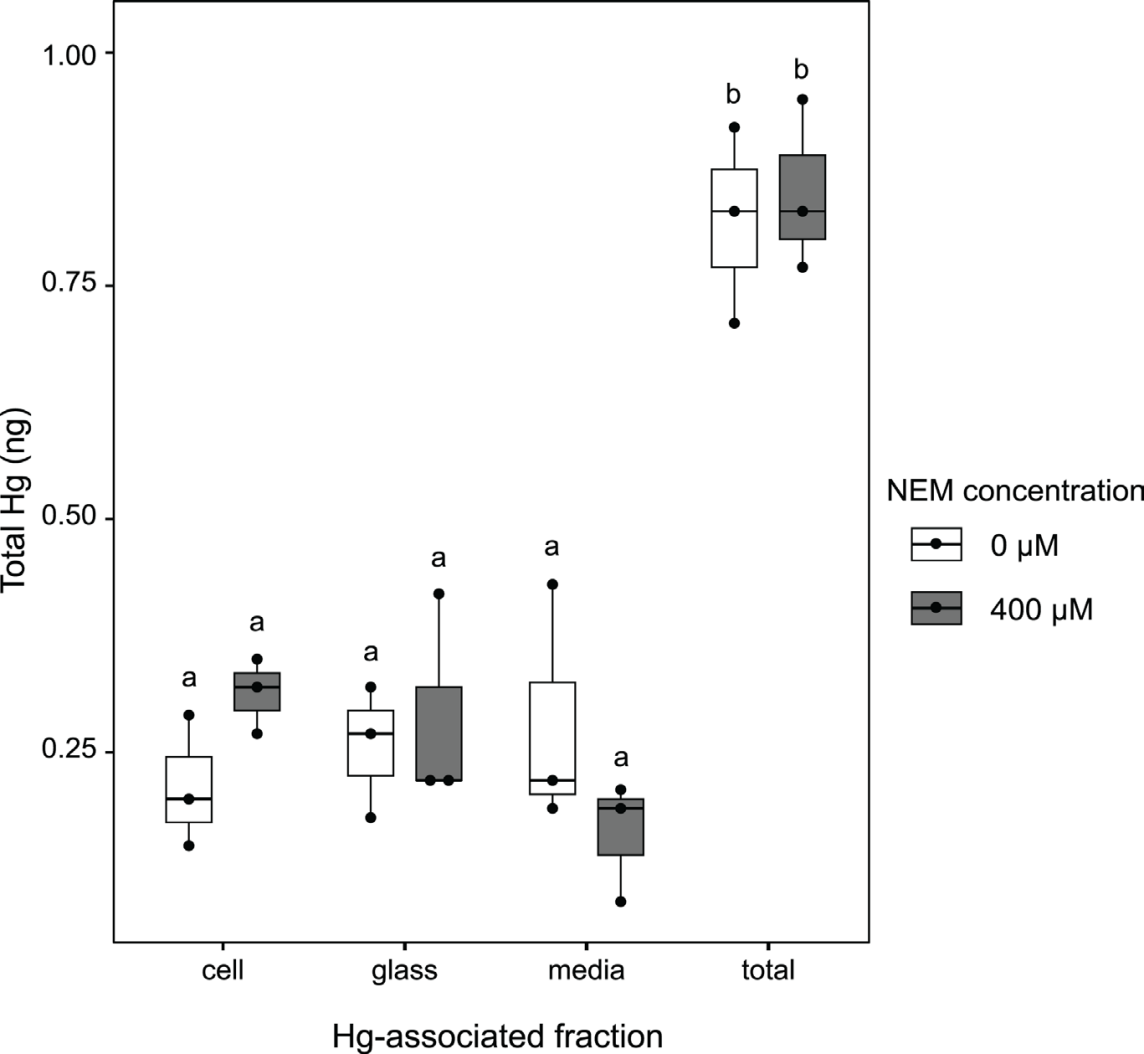
Total Hg quantified from partitioning assays of *E. coli* cultures in medium PYE and exposed to NEM for 3 h. Hg-associated fractions are represented in order from left to right: *E. coli* cell pellet-associated Hg, glass-bound Hg from Balch tubes, Hg remaining in 0.2 µm-filtered spent growth media and the total Hg initially present in the exposure assay. We maintained equal ratios of cell OD 600 nm: Hg: NEM between the exposure assay and bioreactor experiments, i.e. 0.400 OD: 500 pM Hg: 400 µM NEM for these *E. coli* assays and 0.200: 250 pM: 200 µM for *H. modesticaldum* bioreactors. At the start of the exposure, approximately [Hg^II^] = 500 pM or 1 ng in 10 ml of culture was added. The bottom and top of the boxes show the first and third quartiles, respectively; the bar in the middle shows the median, and the whiskers show the minimum and maximum for each treatment. The black circles overlain represent the individual sample measurements for *n*=3 *E*. *coli* cultures. Assumptions of normally distributed residuals (*P*=0.16) and homoscedasticity (*P*=0.99) were met for a two-way parametric ANOVA, which showed there was a significant difference between Hg-associated fractions (*P*=4.8×10^−9^) but not between NEM concentrations (*P*=0.77) or their interaction (*P*=0.248). Letters that are not shared between fractions indicate a significant difference according to the Tukey HSD test (*P*<0.05).

### NEM does not affect Hg partitioning

Considering that Hg^II^ cellular bioavailability is in part influenced by interactions with cell-surface thiol groups [15,43,47], we also aimed to verify if NEM prevented Hg^II^ from associating with cells. To test this, we exposed a non-reducing strain of *E. coli* to Hg^II^ and NEM and analysed to which fractions Hg^II^ was associated after a 3-h incubation. To be consistent with later bioreactor experiments, we used equivalent ratios of cell OD 600 nm: Hg concentration: NEM concentration. As we needed higher cellular biomass to successfully extract cell-bound Hg, we doubled cell OD, Hg and NEM concentrations compared to bioreactors; i.e. for an OD 600 nm of 0.400, we added 500 pM Hg (1 ng in 10 ml) and 400 µM of NEM to these assays.

Here, we report no significant effect of 400 µM NEM addition on the partitioning of Hg to different fractions (*P*=0.77) (Fig. 1). Mean cell-associated total Hg was not different between cells exposed to 0 µM NEM and to 400 µM (0.21±0.07 and 0.31±0.04 ng, respectively). Mirroring these results, there were no significant differences between Hg concentrations remaining in the growth media at 0 and 400 µM NEM (0.28±0.13 and 0.16±0.06 ng, respectively). The per cent mean total Hg recovered was 91±2% across all treatments when comparing the sum of cell-, glass- and media-associated Hg fractions to the Hg initially added to the assays. Our results highlight that picomolar concentrations of Hg^II^ can still partition to cells exposed to 400 µM of NEM. With these results in hand, we proceeded to test the effect of NEM on Hg^II^ reduction by *H. modesticaldum*.

### NEM differentially inhibits Hg^0^ production during photoheterotrophy and fermentation

To test whether NEM addition prevented anaerobic Hg^II^ reduction by *H. modesticaldum* Ice1, we compared cumulative Hg^0^ production over the course of 48 h under both photoheterotrophic and fermentative growth conditions at NEM concentrations of 0, 100 and 200 µM. Here, we added 250 pM of HgCl_2_ to bioreactor cultures containing an equivalent cell density measured as OD 600 nm of 0.2. The sterile growth media bioreactor shows that there is negligible abiotic photoreduction of Hg^II^ in the presence of 100 µM NEM (Fig. S2B).

**Fig. 2.**
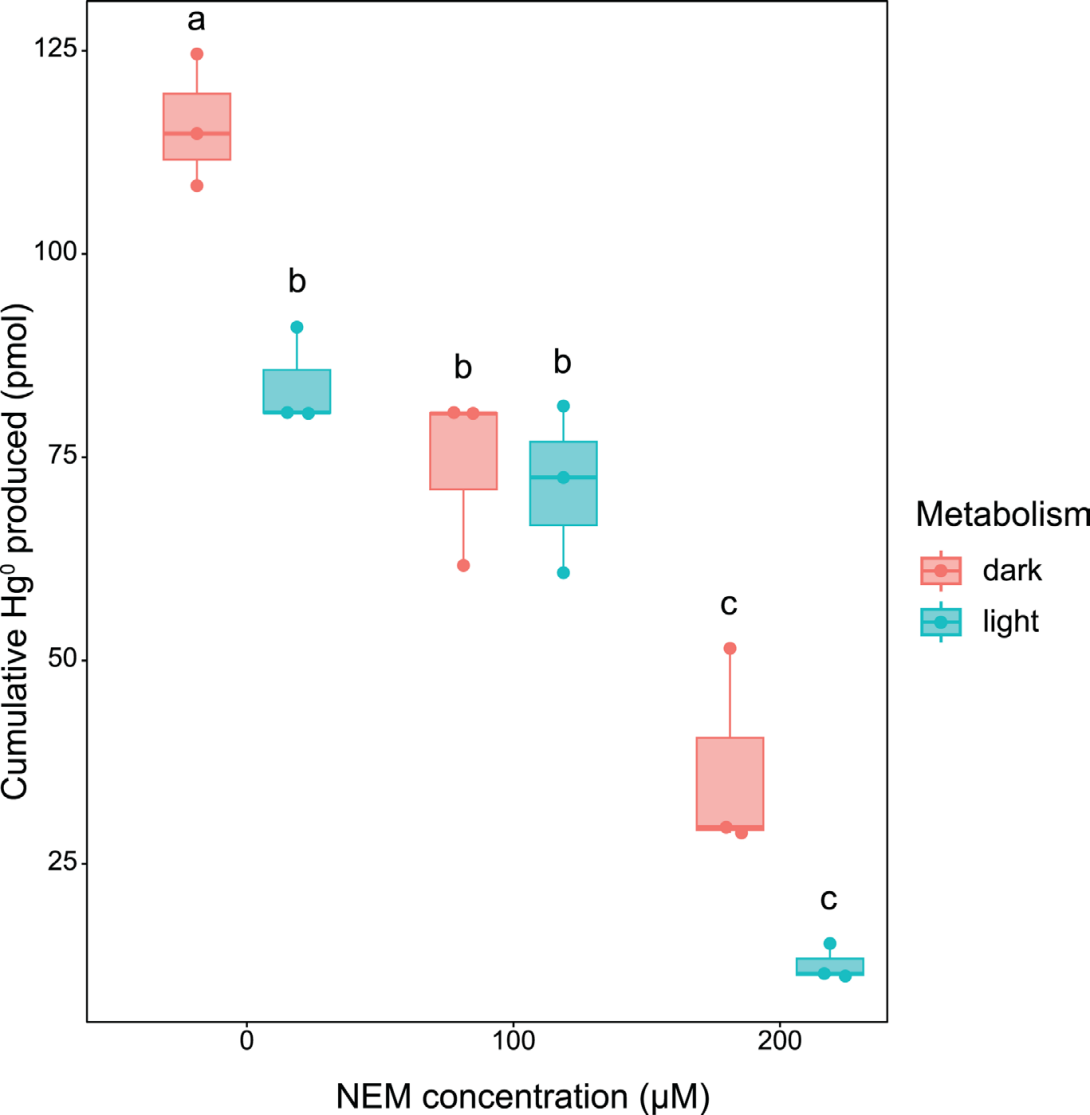
Cumulative Hg^0^ produced during bioreactor experiments with *H. modesticaldum* Ice1 grown photoheterotrophically (light) and fermentatively (dark) in medium PYE exposed to NEM concentrations ranging from 0 to 200 µM. The bottom and top of the boxes show the first and third quartiles, respectively; the bar in the middle shows the median, and the whiskers show the minimum and maximum for each treatment. The circles overlain represent the individual sample measurements. Assumptions of normally distributed residuals (*P*=0.85) and homoscedasticity (*P*=0.93) were met for a two-way parametric ANOVA, which showed there was a significant effect of NEM addition and metabolism on cumulative Hg^0^ production (*P*=2.5×10^−8^ and *P*=6.7×10^−4^, respectively) and a significant effect of their interaction (*P*=0.043). Letters that are not shared between fractions indicate a significant difference according to the Tukey HSD test (*P*<0.05).

**Fig. 3.**
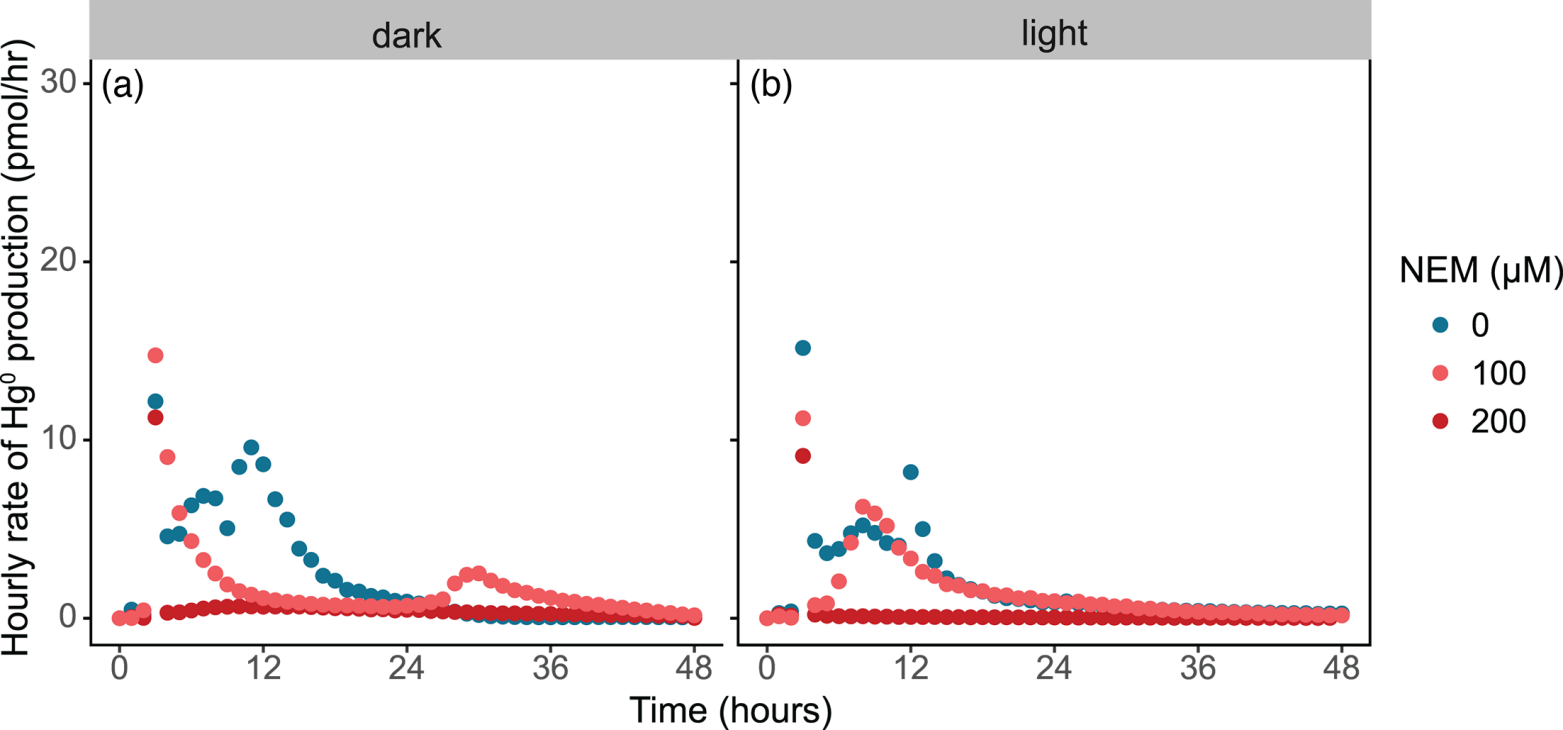
Hourly rate of Hg^0^ production for individual representative bioreactor assays with *H. modesticaldum* Ice1 in medium PYE during (a) dark (fermentative) and (b) light (photoheterotrophic) exposure to NEM.

We found that there was a significant concentration-dependent effect of NEM on cumulative Hg^0^ production by *H. modesticaldum* (*P*=2.5×10^−8^) (Fig. 2). Within the first few hours of bioreactor assays, NEM treatments displayed a large drop in hourly Hg^0^ produced. This drop was most pronounced under fermentative conditions and maximum at 200 µM NEM (Fig. 3). Unlike in Balch tubes, photoheterotrophic and fermentative cultures did not grow in bioreactors exposed to 100 and 200 µM (Fig. S3). We do not attribute the decrease in Hg^0^ production specifically to the lack of growth. Our previous work highlights that Heliobacterial Hg^II^ reduction under phototrophic conditions occurs even in the absence of quantifiable growth or an oxidizable carbon source [9,11]. However, under fermentative conditions, the availability of a carbon source directly impacts the magnitude of Hg^0^ produced [9].

Typically, in the absence of NEM, the magnitude of Hg^II^ reduction is greater for fermentative cultures than for phototrophic ones. To take this difference into account when considering the effect of NEM on Hg^0^ production, we compared the mean per cent difference in Hg^0^ produced between no NEM and NEM-added bioreactors. The phototrophic bioreactors with 200 µM of NEM showed a mean decrease in cumulative Hg^0^ production compared to bioreactors without NEM of 85±2.2% (mean per cent difference ±sem per cent). On the other hand, the fermentative bioreactors with 200 µM of NEM exhibited a lower mean decrease of 68.4±4.5%. Interestingly, this effect is inversed at a concentration of 100 µM where we observe a greater mean decrease in fermentative Hg^0^ production (36±7.6%) than phototrophic Hg^0^ production (14.8±22.8%). It remains unknown why 100 µM NEM had a greater inhibitory effect on fermentative Hg^0^ production.

## Discussion

### Hg partitioning assay indicates that NEM inhibition of Hg^II^ reduction does not result from blocking Hg’s ability to partition into cells

The high affinity of Hg^II^ for thiol ligands means that on a typical cell surface with ligands such as thiols, carboxyls and phosphates, Hg^II^ will first bind to thiol groups, often in two or more thiol coordination [37]. There are both passive [48,49] and active [44,50] Hg^II^ uptake mechanisms; some entail interactions with cell-surface thiols, while others do not. Bacterial surface thiol concentrations can range from 16 to 33 mmol per gramme of wet cell weight [51]. Furthermore, estimated intracellular thiol concentrations for anaerobes range from 10 to 200 mM [52]. As we did not observe significant differences in Hg partitioning in the presence of micromolar levels of NEM, we do not expect all cell-surface and intracellular thiols to be irreversibly bound to this chemical inhibitor, likely leaving millimolar amounts of thiols available for potential Hg^II^ interaction. Therefore, in bioreactor assays with *H. modesticaldum*, it is very unlikely that NEM inhibition of Hg^II^ reduction is caused by binding to all cellular thiols and preventing uptake. However, as we used the Gram-negative *E. coli* for Hg partitioning assays, we worked under the assumption that Hg^II^ bioavailability and cell-surface interactions are comparable to those with the Gram-positive *Heliobacteria*. We chose to work with *E. coli* instead of *Heliobacteria* for this assay because of the risk of results being skewed by Hg^0^ evasion and loss during the multiple steps of the partitioning assays. We cannot discount that their dissimilarities in cell membrane structures may cause differences in interactions between Hg^II^ and cells and between NEM and cell-surface thiols. However, some of these differences may be negligible as cell-surface thiol concentrations of Gram-positive and Gram-negative bacteria and between mesophilic and thermophilic strains are similar [51].

At the onset of the development of Hg partitioning assays with *E. coli*, we sought to quantify intracellular versus cell-surface Hg^II^. Our initial attempt at the assay used the powerful Hg chelator DMSA [30] to remove cell-surface Hg, but it caused the cell pellet to lose integrity and shrink in size after successive cell washing and re-pelleting steps. We suspect that DMSA was destabilizing the outer membranes of *E. coli* and causing cell lysis, an effect reported by others [40]. Ultimately, we could not successfully extract membrane-associated Hg and intracellular-associated Hg. Therefore, we cannot further substantiate whether the concentration-dependent inhibition of NEM on Heliobacterial Hg^II^ reduction is linked to greater extracellular or intracellular Hg partitioning. An alternative chemical inhibitor approach may be used to test if irreversible binding to cell-surface thiols blocks all Hg^II^ reduction. Monobromo(trimethylammonio)bimane (mBBr) binds irreversibly to thiols while being too large to penetrate cell membranes [53,54], unlike NEM, which can reach the cytosol [55,56]. In future work, we plan to test Hg^II^ reduction by *H. modesticaldum* under mBBr exposure.

### NEM may inhibit essential enzymatic pathways

NEM that enters the cytosol and irreversibly binds to thiol groups may prevent multiple cellular pathways from operating normally, including those linked to Hg^II^ reduction. Research using NEM as an alkylating agent has highlighted its inhibitory effect on multiple proteins and intracellular thiol-bearing molecules. For those relevant to *Heliobacteria*, we discuss their potential role in Hg^II^ reduction.

In *H. modesticaldum*, the photosynthetic reaction centre and PFOR reduce ferredoxins, one-electron cytosolic redox cofactors [25]. Ferredoxins carry electrons to other oxidoreductases, namely, to the NADH-dependent ferredoxin:NADP+ oxidoreductase (NfnAB) [25]. This type of oxidoreductase, first characterized in *Clostridium kluyveri*, shares sequence and functional similarity with plant FNR [57]. NfnAB is an electron-bifurcating enzyme that couples the exergonic reduction of NADP^+^ with ferredoxin and the endergonic reduction of NADP^+^ with NADH in a reversible reaction [58]. As such, it plays a crucial role in maintaining redox homeostasis by balancing the ratio of oxidized and reduced pyridines [i.e. NAD(H) and NADP(H)] and ferredoxin pools [59]. Considering that electron flow, or reducing power, from fermentation and phototrophy both interface via NfnAB and the enzyme catalyses 2-electron transfer reactions, it may be of interest for future investigations. NfnAB may either be the enzymatic site of Hg^II^ reduction or the next oxidoreductase controlling electron flow towards a yet unidentified cellular site of Hg^II^ reduction. Though Hg^II^’s affinity for bacterial NfnAB is not documented, there is precedent to suspect Hg^II^-thiol interactions on this protein. A study investigating cadmium inhibition of spinach FNR activity found that cadmium was binding to cysteine residues on FNR, which was further confirmed by showing decreased binding of Cd^II^ to FNR upon the addition of NEM [60,61]. The authors further suggest that cadmium’s inhibition of FNR may cause conformational changes that disturb ferredoxin binding and thus electron transfer. As Hg^II^ and Cd^II^ share chemical properties (e.g. cationic soft metals having an affinity for sulphur and the ability to displace Zn and Fe in metalloproteins [62]) and plant FNR and prokaryotic NfnAB share sequence similarity, Hg^II^ may be able to interact with Heliobacterial NfnAB and perhaps intercept electrons. Our current methodology cannot verify whether NEM inhibited NfnAB activity and whether this, in turn, inhibited Hg^II^ reduction. Given the central role of NfnAB in *H. modesticaldum,* no gene deletion attempt has been successful to date. A deletion approach may thus not be an achievable way to test the role of this enzyme in Hg^II^ reduction. Gene insertions into non-reducing anaerobes or *in vitro* purified protein assays may be alternative options to test whether NfnAB and its cofactors can catalyse Hg^II^ reduction.

### NEM may affect cytosolic thiol-based redox buffers and other redox regulation mechanisms

Strong irreversible binding of NEM to thiols may stop the proper functioning of cytosolic low molecular mass (LMM) redox buffers like free cysteine, glutathione [56] and bacillithiols, which *H. modesticaldum* uses in lieu of glutathione [63]. Upon entering the cytosol, Hg^II^ will likely interact with intracellular thiol-based redox buffers such as bacillithiols and free cysteines and quench or possibly oxidize them [64,65]. Redox buffers offer intracellular protection against the oxidative stress of Hg by being themselves a target for oxidation [66]. Thiol-based redox homeostasis systems, such as thioredoxins/thioredoxin reductases and bacilliredoxins/bacillithiol disulphide reductases, work to reduce oxidized protein thiols and bacillithiols, respectively [67,69]. We propose the reduction to Hg^0^ may happen via cellular re-reduction of Hg^II^ bound to thiols via such thiol-based redox-regulating machinery. A similar mechanism has been proposed but not verified in *Thermus thermophilus* HB27 wherein thioredoxins might directly reduce or sequester Hg^II^, or that thioredoxin reductases may reduce thioredoxin-Hg^II^ complexes, thus leading to detoxification by evasion of Hg^0^ [70]. Future experiments targeting these proteins via either inhibitory or gene deletion approaches may serve to elucidate whether there is a coupling between thiol-based redox homeostasis systems and anaerobic Hg^II^ reduction.

There is little doubt that NEM lacks enzymatic specificity due to its irreversible binding mechanism to thiols. However, our findings support a reduction pathway where Hg^II^ interacts with thiol-bearing cell machinery. It is yet to be confirmed whether this interaction occurs with cell-surface thiols, as part of Hg^II^ uptake, or with thiol-bearing intracellular proteins and LMM molecules. With the recent advent of a reliable CRISPR-based molecular biology toolkit for *H. modesticaldum* [25,71,74], it is now possible to delete or insert specific genes of interest to resolve mechanistic details for anaerobic Hg^II^ reduction. These experiments will be important for identifying genetic controls in *Heliobacteria* and can eventually be used to develop genetic targets to track anaerobic Hg^II^ reduction in the environment.

## Supplementary material

10.1099/acmi.0.000932.v3 Uncited Supplementary Material 1.

10.1099/acmi.0.000932.v3 Uncited Table S1.

## Abbreviations

DMSA: dimercaptosuccinic acid
FNR: ferredoxin:NADP+ oxidoreductase
GMM: glucose minimal medium
HSD: honest significant difference
LMM: low molecular mass
mBBr: monobromo(trimethylammonio)bimane
MMHg: monomethylmercury
NEM: *N*-ethylmaleimide
NfnAB: NADH-dependent ferredoxin:NADP+ oxidoreductase
pETC: photosynthetic electron transport chain
PFOR: pyruvate:ferredoxin oxidoreductase
