# Supplementary material for: Anaerobic HgII reduction is driven by cellular HgII-thiol interactions
Source: Access Microbiol. 2025 Jan 28;7(1):000932.v3. doi: 10.1099/acmi.0.000932.v3 (PMC12282025; doi:10.1099/acmi.0.000932.v3)
Supplement: Uncited Supplementary Material 1. [file acmi-7-00932-s001.pdf]

SUPPLEMENTARY FIGURES

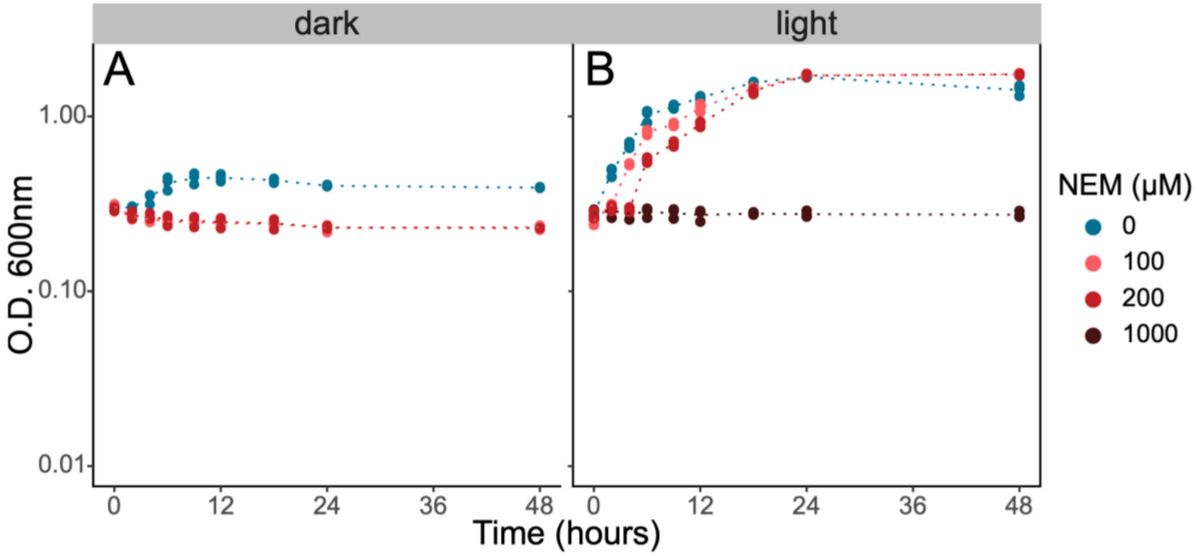

SI Figure 1. Minimum inhibitory concentration assays of *H. modesticaldum* Ice1 exposed to a gradient of NEM concentrations for A) fermentative (dark) cultures and B) photoheterotrophic (light) cultures. For each concentration, biological triplicate cultures were prepared to an initial O.D. of ~0.2 in Balch tubes containing 10 mL of medium PYE and O.D. 600 nm was measured over time to assess growth.

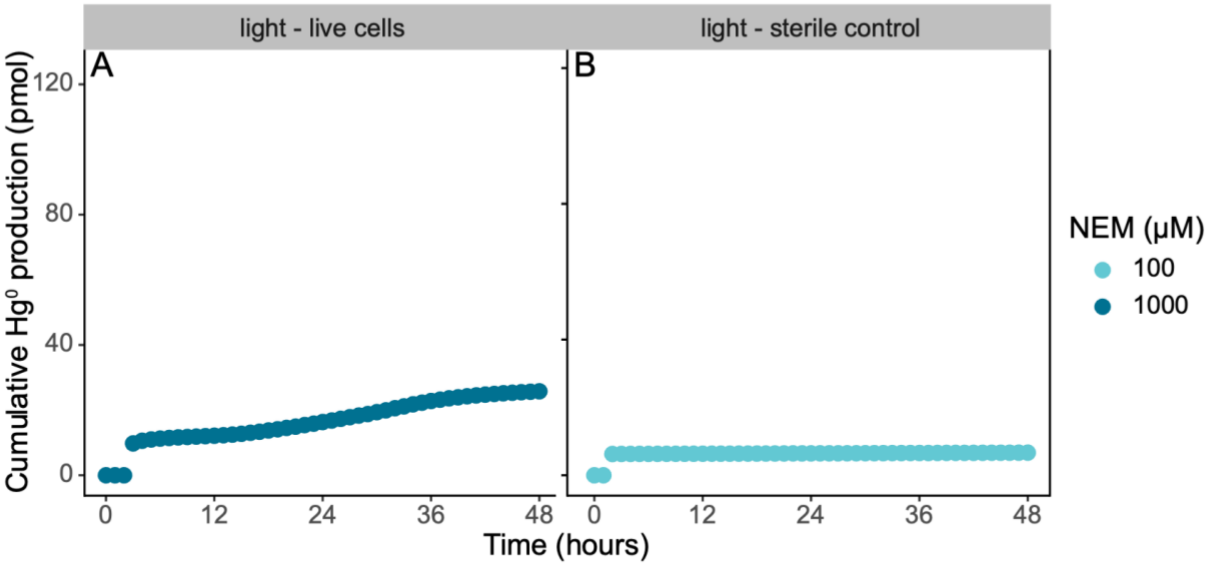

SI Figure 2. Hourly cumulative Hg^0 produced for bioreactor experiments with A) live photoheterotrophic (light) cultures of *H. modesticaldum* exposed to 1000 µM of NEM and B) sterile growth medium in the light devoid of cells and exposed to 100 mM.

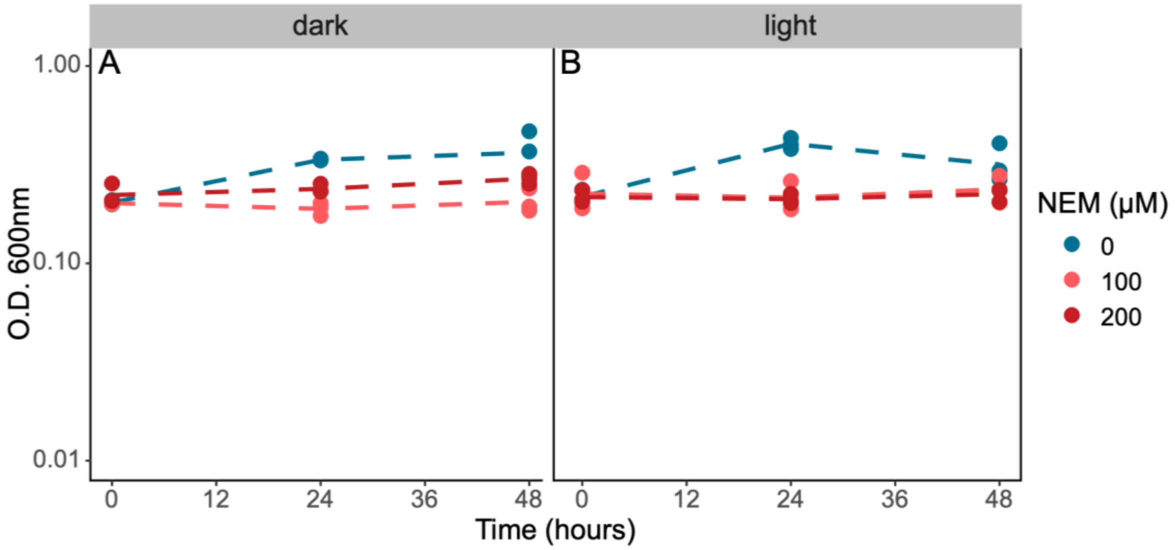

SI Figure 3. Measured growth via optical density (O.D. 600nm) of *H. modesticaldum* cultures during bioreactor experiments. A) growth of fermentative (dark) cultures and B) growth of photoheterotrophic (light) cultures. One measurement per bioreactor assay and per timepoint was taken.
